# Increased regulatory B cells are involved in immune evasion in patients with gastric cancer

**DOI:** 10.1038/s41598-019-49581-4

**Published:** 2019-09-11

**Authors:** Yuki Murakami, Hiroaki Saito, Shota Shimizu, Yusuke Kono, Yuji Shishido, Kozo Miyatani, Tomoyuki Matsunaga, Yoji Fukumoto, Keigo Ashida, Tomohiko Sakabe, Yuji Nakayama, Yoshiyuki Fujiwara

**Affiliations:** 1 Division of Surgical Oncology, Department of Surgery, School of Medicine, Tottori University Faculty of Medicine, 36-1 Nishi-cho, Yonago, 683-8504 Japan; 2 Division of Organ Pathology, Department of Pathology, Faculty of Medicine, Tottori University, 86 Nishi-cho, Yonago, 683-8503 Japan; 3 Division of Radioisotope Science, Research Initiative Center, Organization for Research Initiative and promotion, Tottori University, 86 Nishi-cho, Yonago, 683-8503 Japan

**Keywords:** Gastric cancer, Tumour immunology

## Abstract

Accumulating evidence has indicated that immune regulatory cells are involved in the establishment of tumoral immune evasion. However, the role of regulatory B cells (Bregs) in this remains unclear. Here, we identified a role for Bregs in immune evasion in gastric cancer (GC) patients. The frequency of peripheral Bregs was significantly higher in GC patients than in healthy controls (*P* = 0.0023). Moreover, the frequency of CD19^+^CD24^hi^CD27^+^ B cells in GC tissue was significantly higher than in peripheral blood and healthy gastric tissue. Carboxyfluorescein succinimidyl ester labeling revealed that CD19^+^CD24^hi^CD27^+^ B cells could suppress the proliferation of autologous CD4^+^ T cells. Moreover, CD19^+^CD24^hi^CD27^+^ B cells inhibited the production of interferon-gamma by CD4^+^ T cells. Double staining immunohistochemistry of interleukin-10 and CD19 revealed 5-year overall survival rates of 65.4% and 13.3% in Breg^Low^ and Breg^High^ groups, respectively (*P* < 0.0001). Multivariate analysis indicated that the frequency of Bregs was an independent prognostic indicator in GC patients. Taken together, our results show the existence of Bregs in GC tissue, and indicate that they are significantly correlated with the prognosis of GC patients.

## Introduction

Gastric cancer (GC) is one of the most common malignancies and ranks second among all cancer-related deaths worldwide^1^. Recently, it was shown that the antibody to programmed cell death protein 1, known as the immune checkpoint inhibitor, is effective in the treatment of unresectable advanced and recurrent GC^2^. This clearly indicates that effective immunity to GC cells can be induced in GC patients, which improves their prognosis.

The expression of tumor rejection antigens such as MAGE 1–3 and the presence of tumor-specific cytotoxic T cells have been reported in GC patients^3,4^, but the spontaneous rejection of established GC is rarely seen. Furthermore, the efficacy of immune checkpoint inhibitors is still limited^2^, possibly because of tumoral immune evasion. Some mechanisms responsible for tumoral immune evasion in GC patients include the production of immune suppressive cytokines, such as interleukin (IL)-10 and transforming growth factor-beta 1, by tumor cells and other cells exist at the tumor microenviroment^5,6^, and the impaired function of immune cells including CD8+ T cells and natural killer cells^7^. The presence of immune regulatory cells is also important in tumoral immune evasion. One of the most characterized immune regulatory cells are regulatory T cells (Treg), whose levels are increased in both peripheral blood and tumor tissue of GC patients where they are associated with tumoral immune evasion^8^.

B lymphocytes play a central role in humoral immunity through the production of immunoglobulin. Recently, a subset of B cells, regulatory B cells (Bregs), was demonstrated to exert immunoregulatory functions through production of the immunosuppressive cytokine IL-10^9^. Although most Breg studies have focused on autoimmune mouse models and patients with autoimmune diseases, several mouse cancer studies have also revealed the existence of protumorigenic Bregs^10–12^. At present, however, the subset composition and function of Bregs in human cancer including GC are largely unclear. Therefore, we undertook the present study to evaluate Bregs in GC to assess possible mechanisms for immune evasion by Bregs.

## Results

### Bregs in peripheral blood

**Figure 1:**
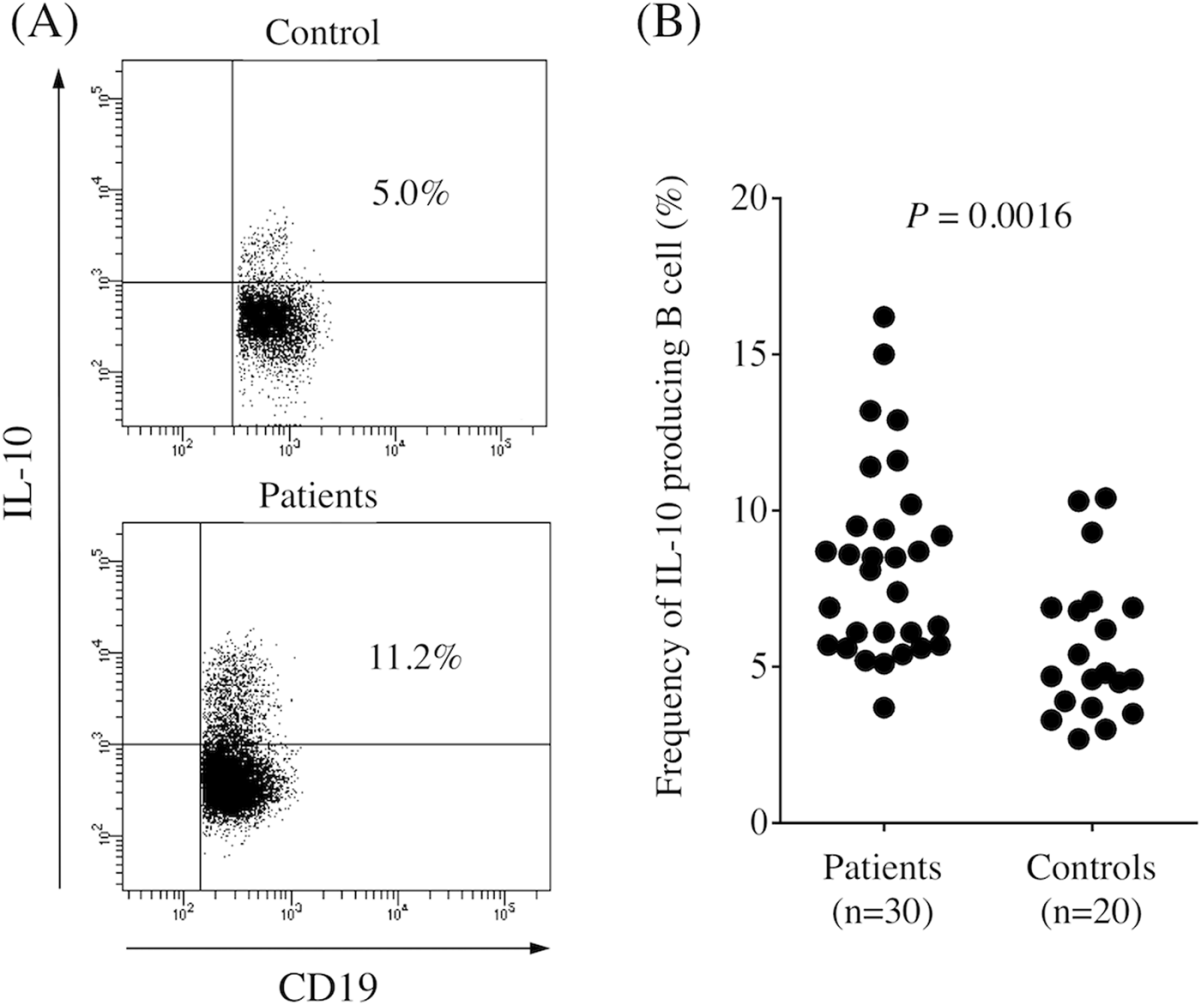
(**A**) Representative image of regulatory B cells in control and gastric cancer patients. (**B**) Percentage of CD19^+^IL-10^+^cells among CD19^+^ B cells in peripheral blood obtained from gastric cancer patients and healthy controls. The frequency of Bregs was significantly higher in gastric cancer patients than in healthy controls (*P* = 0.0016).

**Figure 2:**
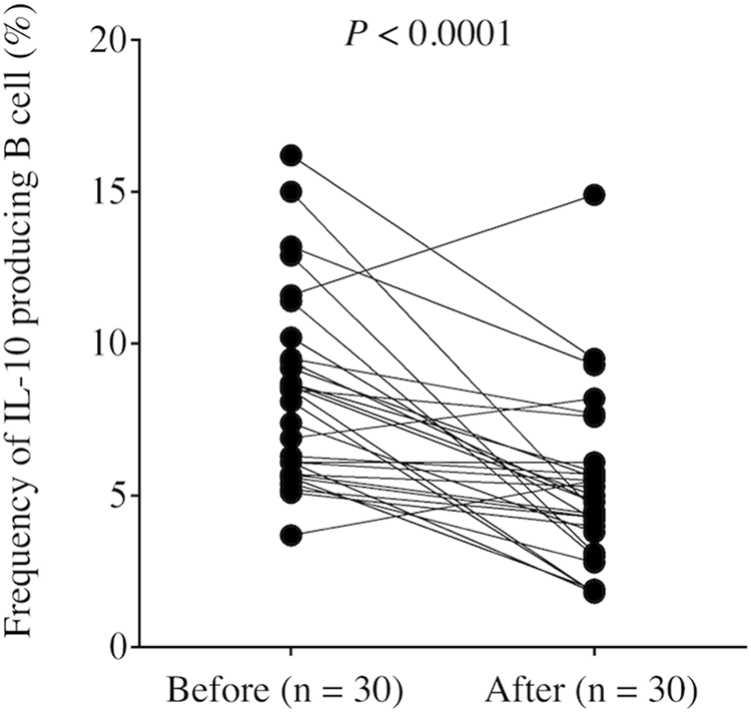
The frequency of Bregs in peripheral blood was significantly decreased 1 month after surgery compared with before the operation (*P* < 0.0001).

Bregs exert important immunoregulatory functions through production of the immunosuppressive cytokine IL-10, so we first determined the frequency of IL-10-producing B cells (Bregs) in the peripheral blood obtained from controls and GC patients. The frequency of Bregs was significantly higher in GC patients than in controls (Fig. 1, *P* = 0.0016). Table 1 shows that there was no significant correlation between the frequency of Bregs and clinicopathologic characteristics. We found that the frequency of Bregs significantly decreased 1 month after surgery compared with prior to the operation (Fig. 2, *P* < 0.0001).

**Table 1:** Frequency of peripheral Bregs and clinicopathological variables in gastric cancer patients.

Variable	Bregs (%)	*P* value
Age		0.175
<75 (n = 21)	8.77 ± 0.71	
≥75 (n = 9)	7.04 ± 0.93	
Sex		0.973
Male (n = 21)	8.27 ± 0.67	
Female (n = 9)	8.22 ± 1.19	
Histology^a^		0.484
Differentiated (n = 15)	7.84 ± 0.64	
Undifferentiated (n = 15)	8.67 ± 0.97	
Depth of invasion^b^		0.121
T1 (n = 17)	9.04 ± 0.82	
T2/3/4 (n = 13)	7.22 ± 0.74	
Lymph node metastasis		0.995
Absent (n = 18)	8.25 ± 0.69	
Present (n = 12)	8.26 ± 1.05	
Lymphatic invasion		0.554
Absent (n = 12)	7.83 ± 0.77	
Present (n = 18)	8.54 ± 0.83	
Vascular invasion		0.473
Absent (n = 16)	8.65 ± 0.78	
Present (n = 14)	7.80 ± 0.88	
Stage		0.498
I (n = 19)	8.56 ± 0.76	
II/III/IV (n = 11)	7.73 ± 0.89	

^a^Differentiated, papillary, or tubular adenocarcinoma; undifferentiated, poorly differentiated, or mucinous adenocarcinoma, or signet-ring cell carcinoma.

^b^Depth of invasion: T1, tumor invasion of the lamina propria or submucosa; T2, tumor invasion of the muscularis propria; T3, tumor invasion of the subserosa; T4, tumor penetration of the serosa or tumor invasion of adjacent organs.

All results are expressed as the mean ± SD.

### Surface markers of Bregs

**Figure 3:**
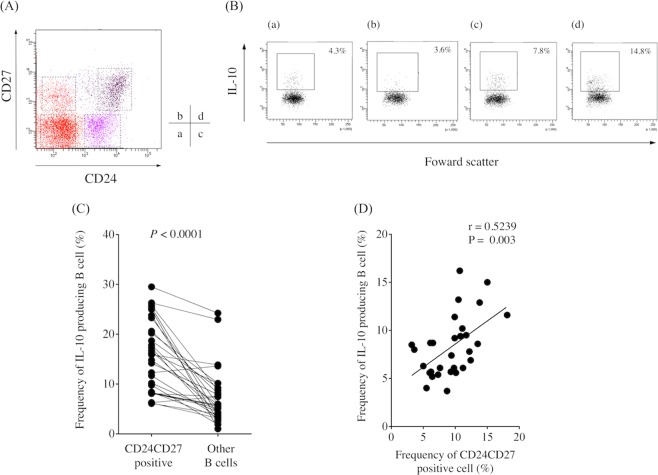
(**A**) Representative image of the frequency of regulatory B cells according to CD24 and CD27 expression in gastric cancer patients. (**B**) The frequency of IL-10-producing B cells in (a) CD19^+^CD24^-^CD27^-^ B cells, (b) CD19^+^CD24^-^CD27^+^ B cells, (c) CD19^+^CD24^+^CD27^−^ B cells, and (d) CD19^+^CD24^hi^CD27^+^ B cells. (**C**) The frequency of IL-10-producing B cells was significantly higher in the CD19^+^CD24^hi^CD27^+^ B cell subset than in other subsets (*P* < 0.0001). (**D**) A significant positive correlation was detected between the frequency of IL-10-producing B cells and the frequency of CD19^+^CD24^hi^CD27^+^ B cells (*r* = 0.5309, *P* = 0.0021).

We examined the expression of surface markers, including CD25, CD39, CD73, CD80, and CD86, in IL-10-producing B cells and non-IL-10-producing B cells and found no difference between them (Supplementary Fig. 1). Previous studies demonstrated that human Bregs were contained in the CD19^+^CD24^hi^CD27^+^ B cell subset^13^. In this study, flow cytometry analysis revealed that the CD19^+^CD24^hi^CD27^+^ B cell subset existed in PBMCs obtained from GC patients (Fig. 3A). Moreover, the frequency of IL-10-producing B cells was significantly higher in the CD19^+^CD24^+^CD27^+^ B cell subset than in other subsets (Fig. 3B,C). IL-10-producing B cells were also significantly higher in number in the CD19^+^CD24^+^CD27^+^ B cell subset than in the CD19^+^CD24^+^CD27^−^ B cell subset (Supplementary Fig. 2). Furthermore, we detected a significant positive correlation between the frequency of IL-10-producing B cells and the frequency of CD19^+^CD24^+^CD27^+^ B cells, indicating that Bregs were enriched in the CD19^+^CD24^+^CD27^+^ B cell subset in GC patients (Fig. 3D).

### Bregs in GC tissue

**Figure 4:**
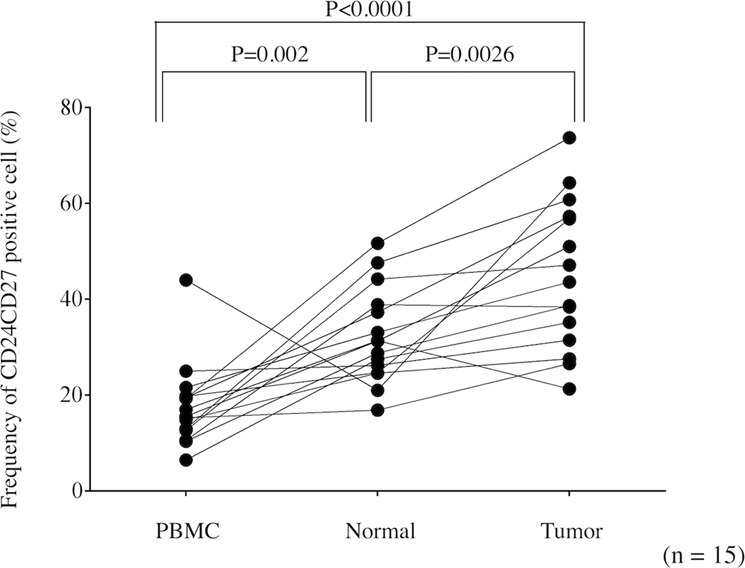
The frequency of the CD19^+^CD24^hi^CD27^+^ B cell subset among CD19^+^ B cells in peripheral blood, healthy gastric tissue, and cancer tissue. The frequency of CD19^+^CD24^hi^CD27^+^ B cells among CD19^+^ B cells was significantly higher in gastric cancer tissue than in peripheral blood and healthy gastric tissue.

We previously demonstrated that immune suppression was stronger in the tumor site than in the peripheral blood of GC patients^7,14,15^. Therefore, to determine if the same phenomenon occurs regarding Bregs, we obtained PBMCs, normal gastric mucosa, and GC tissue from the same patients and determined the frequency of CD19^+^CD24^hi^CD27^+^ B cells in peripheral blood, normal gastric tissue, and GC tissue. The frequency of CD19^+^CD24^hi^CD27^+^ B cells was significantly higher in GC tissue than in peripheral blood and healthy gastric tissue (Fig. 4), indicating that CD19^+^CD24^hi^CD27^+^ B cells containing Bregs are abundant in GC tissue.

### The CD19^+^CD24^hi^CD27^+^ B cell population exerts immunoregulatory functions

**Figure 5:**
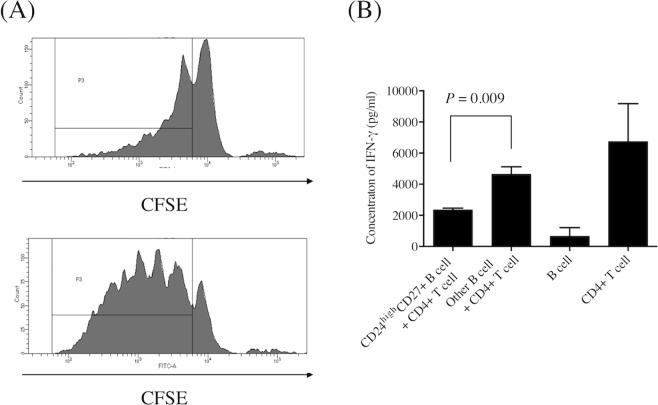
(**A**) Carboxyfluorescein succinimidyl ester cell division assay. The proliferation of CD4^+^ T cells co-cultured with CD19^+^CD24^hi^CD27^+^ B cells (upper panel) was lower than that of CD4^+^ T cells co-cultured with other B cells (lower panel). Image is representative of three experiments. The gate P3 indicates proliferated CD4^+^ T cells. (**B**) Enzyme-linked immunosorbent assay showing that IFN-gamma production by CD4^+^ T cells co-cultured with CD19^+^CD24^hi^CD27^+^ B cells was significantly lower than that of CD4^+^ T cells co-cultured with other B cells (n = 3).

The CFSE experiment revealed that CD19^+^CD24^hi^CD27^+^ B cells were able to suppress the proliferation of autologous CD4^+^ T cells (Fig. 5A). Moreover, ELISA demonstrated that CD19^+^CD24^hi^CD27^+^ B cells inhibited the production of IFN-gamma by autologous CD4^+^ T cells (Fig. 5B). We also confirmed that CD19^+^CD24^hi^CD27^+^ B cells inhibited IFN-gamma production by autologous CD4^+^ T cells using flow cytometry (Supplementary Fig. 3). However, apoptosis assay revealed that CD19^+^CD24^hi^CD27^+^ B cells have no effect on apoptosis of CD4^+^ T cells (Supplementary Fig. 4). These results clearly demonstrated that the CD19^+^CD24^hi^CD27^+^ B cell population contains cells that exert important immunoregulatory functions.

### Bregs worsen the prognosis of GC cancer patients

**Figure 6:**
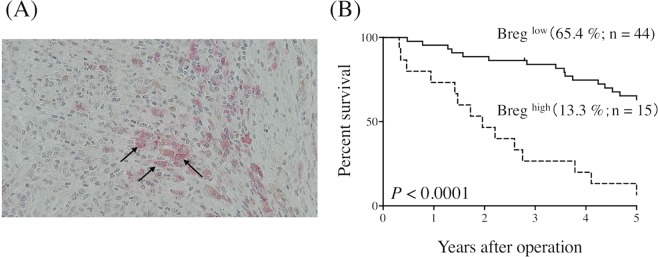
(**A**) Representative image of regulatory B cells (arrows) in gastric cancer tissue following double staining immunohistochemistry (pink, CD19; brown, IL-10; magnification 400×). (**B**) The prognosis of patients with a marked infiltration of regulatory B cells was significantly worse than that of those with a slight infiltration of regulatory B cells (*P* < 0.0001).

Finally, we determined the correlation between the frequency of Bregs and prognosis in GC patients. To this end, we performed double staining immunohistochemistry of IL-10 and CD19 (Fig. 6A). The frequency of Bregs, represented by the ratio of the number of cells positive for both CD19 and IL-10 to the number of cells positive for CD19, was 13.9 ± 7.8%. There was no significant correlation between the frequency of Bregs and clinicopathologic characteristics (Table 2). ROC analysis indicated an optimal cutoff value of 19.35% for the highest Youden index. Based on this, patients were divided into Breg^Low^ (<19.35%) and Breg^High^ (≥19.35%) groups. Five-year OS rates were significantly different between the two groups, at 65.4% and 13.3%, respectively (*P* < 0.0001, Fig. 6B). Univariate analysis indicated that tumor size, lymph node metastasis, lymphatic invasion, and the frequency of Bregs were significantly associated with OS (Table 3). Finally, multivariate analysis indicated that the frequency of Bregs was an independent prognostic indicator of GC patient survival, along with lymph node metastasis (Table 3).

**Table 2:** Frequency of tumor-infiltrating Bregs and clinicopathological variables in gastric cancer patients.

Variable	Bregs (%)	*P* value
Age		0.09
<75 (n = 40)	12.8 ± 7.73	
≥75 (n = 19)	16.4 ± 7.49	
Sex		0.11
Male (n = 36)	15.2 ± 8.15	
Female (n = 23)	11.9 ± 6.87	
Histology^a^		0.876
Differentiated (n = 21)	14.2 ± 7.12	
Undifferentiated (n = 38)	13.8 ± 8.22	
Depth of invasion^b^		0.479
T1/2 (n = 20)	12.9 ± 7.71	
T3/4 (n = 39)	14.5 ± 7.88	
Lymph node metastasis		0.09
Absent (n = 17)	11.2 ± 5.88	
Present (n = 42)	15.0 ± 8.25	
Lymphatic invasion		0.181
ly 0/1 (n = 23)	12.2 ± 6.39	
ly 2/3 (n = 36)	15.0 ± 8.47	
Vascular invasion		0.322
v 0/1 (n = 31)	14.9 ± 8.26	
v 2/3 (n = 28)	12.9 ± 7.22	
Stage		0.125
I /II (n = 28)	12.3 ± 7.20	
III/IV (n = 31)	15.4 ± 8.11	

See Table 1 for details of histology and depth of invasion.

All results are expressed as the mean ± SD.

**Table 3:** Univariate and multivariate analysis of factors prognostic for overall survival in patients with GC.

	Univariate analysis			Multivariate analysis		
	Hazard ratio	95% CI	*P* value	Hazard ratio	95% CI	*P* value
Gender (male vs. female)	0.565	0.285–1.12	0.1			
Age^a^	1.025	0.995–1.06	0.1			
Histology (undifferentiated vs. differentiated)	1.018	0.499–2.06	0.96			
Tumor size^a^	1.122	1.04–1.22	0.005	1.052	0.952–1.16	0.32
Depth of invasion (T2 – T4)	1.305	0.849–2.01	0.23			
Lymph node metastasis (N0 – N3)^b^	1.982	1.43–2.75	0.0005	2.022	1.43–2.86	0.0006
Lymphatic invasion (Ly0 – Ly3)^c^	1.772	1.14–2.77	0.012	1.063	0.627–1.80	0.82
Venous invasion (V0 – V3)^d^	0.973	0.639–1.48	0.9			
Regulatory B cell (Breg^High^ vs. Breg^Low^)	5.012	2.40–10.45	<0.0001	4.985	2.36–10.54	<0.0001

^a^Continuous variables

^b^N0: no regional lymph node metastases; N1: Metastasis in 1–2 regional lymph nodes; N2: Metastasis in 3–6 regional lymph nodes; N3; Metastasis in ≥7 regional lymph nodes.

^c^Lymphatic invasion: Ly0–Ly3, grade of lymphatic invasion.

^d^Venous invasion: V0–V3, grade of venous invasion.

See Table 1 for details of histology and depth of invasion.

CI: confidence interval.

## Discussion

An increase in immune cells with negative immune regulatory functions, including Tregs, myeloid-derived suppressor cells, and CD4^+^NKG2D^+^ T cells, has been demonstrated in cancer patients. Of importance is that these immune regulatory cells impaired host immunity against cancer cells^16–20^, resulting in tumor progression and poor prognosis. In the current study, we found that IL-10-producing Bregs, another type of immunosuppressive cell, were increased in the peripheral blood of GC patients compared with controls, indicating that Bregs are closely associated with immune evasion in GC.

One of the most important issues of Bregs research is identifying markers. In the case of mice, Evans *et al*. defined Bregs as CD19^+^CD21^hi^CD23^hi^IgM^hi^CD24^hi ^^21^, while Yanaba *et al*. demonstrated that they were contained in the CD19^+^CD5^+^CD1d^hi^ population^22^. However, although these populations contained Bregs, the phenotyping strategies did not uniquely identify them. In the case of human Bregs, Blair *et al*. demonstrated that human CD19^+^CD24^hi^CD38^hi^ B cells, which normally defines human transitional B cells, exhibit regulatory capacity through the production of IL-10 in healthy individuals^23^. Furthermore, Iwata *et al*. demonstrated that Bregs were predominantly found within the CD19^+^CD24^hi^CD27^+^ B-cell subpopulation^13^. However, as for mouse Bregs, no phenotype has been identified to uniquely identify human Bregs to date.

In the present study, we confirmed that the CD19^+^CD24^hi^CD27^+^ B cell population contained cells that suppressed the proliferation of CD4^+^ T cells and inhibited the secretion of IFN-gamma by autologous CD4^+^ T cells, indicating that the CD19^+^CD24^hi^CD27^+^ B cell population contained Bregs. However, the frequency of Bregs was not high even in the CD19^+^CD24^hi^CD27^+^ B cell subset, and Bregs were also contained in other populations, indicating that this phenotyping strategy could not uniquely identify Bregs. Therefore, other identifying strategies are urgently required to progress Bregs research.

We also found that the frequency of Bregs significantly decreased 1 month after surgery, and that CD19^+^CD24^hi^CD27^+^ B cells containing Bregs were significantly higher in GC tissue than in peripheral blood and healthy gastric tissue. This indicated that some cancer cell factors might affect the number of Bregs. In this regard, Olkhanud *et al*. demonstrated that the intraperitoneal injection of supernatant obtained from the culture of tumor cells could promote Breg accumulation, indicating that the secretion of soluble factors by tumor cells is responsible for increases in Breg^24^. Although the detailed mechanisms of Breg increases in GC patients remain unclear, it is likely that they are similar to those observed in the mouse model.

With regard to the correlation between Bregs and cancer progression, increased numbers of Treg cells were shown to be related to poor survival in patients with hepatocellular carcinoma^25^ and ovarian cancer^16^. This shows that immune suppression is closely associated with tumor progression. In support of this, we found that increased numbers of Bregs were significantly correlated with poor prognosis in GC patients although there was no significant correlation between the frequency of Bregs and clinicopathologic characteristics in the current study. In this regard, recurrence usually arises from micrometastases that cannot be detected by ordinary diagnostics such as ultrasonography, computed tomography, and positron emission tomography. It is likely that immune suppression by Bregs induces the growth of residual cancer cells, which eventually results in recurrence after operation. To the best of our knowledge, ours is the first demonstration of a significant correlation between Bregs and prognosis in GC.

The close correlation between Bregs and prognosis suggests that Bregs are attractive targets for GC treatment. Olkhanud *et al*. demonstrated that Bregs, defined as CD19^+^CD25^+^B220^+^ B cells, were closely linked with cancer progression in the mouse mammary adenocarcinoma 4T1 cancer model^24^. Importantly, injections of an anti-B220 antibody decreased the B220 population in lymph nodes and spleens of this model, resulting in the significant decrease of lung metastases during a mammary carcinoma challenge. This clearly indicated that targeting Bregs was effective for the treatment of cancer. It was also recently demonstrated that Bregs express programmed death-ligand 1, which is closely associated with immunoregulatory function^26^, and it is likely that they express other molecules with similar functions. Immunotherapy that targets these molecules might therefore provide a breakthrough in the treatment of GC. Further studies are required to determine the potential of targeting Bregs in the treatment of GC.

In conclusion, Bregs exist in GC tissue and were significantly correlated with the prognosis of GC patients. Immunotherapy that targets Bregs could therefore provide a novel form of GC treatment.

## Methods

### Patients and healthy donors

Thirty patients (21 males and 9 females; mean age, 67.8 ± 13.4 years), treated at Tottori University Hospital (Yonago, Japan) and pathologically diagnosed with gastric adenocarcinoma, were enrolled in this study. None of the patients had received radiotherapy, chemotherapy, or other medical interventions before surgery. The study protocol was approved by the Institutional Review Board at Tottori University Hospital. All procedures followed were in accordance with the ethical standards of the responsible committee on human experimentation (institutional and national) and with the Helsinki Declaration of 1964 and later versions. Informed consent was obtained from all participants for inclusion in the study. Healthy age-matched controls (n = 20; 14 males and 6 females; mean age, 68.8 ± 11.4 years) were also recruited. Immunohistochemical analysis was performed using paraffin-embedded GC samples from 59 patients who underwent gastrectomy at Tottori University Hospital from 2003 to 2011. Clinicopathologic findings were determined according to the Japanese classification of GC^27^.

### Preparation of peripheral blood mononuclear cells (PBMCs)

Heparinized blood was obtained from patients and controls, and PBMCs were isolated by centrifugation using a Ficoll–Paque (Pharmacia, Uppsala, Sweden) gradient.

### Isolation of tumor-infiltrating lymphocytes

Fresh tumor tissues and normal gastric mucosa obtained from a site in the resected specimen that was as far away as possible from the tumor were washed with RPMI-1640 medium (Lonza, Basel, Switzerland), cut into small pieces, and digested with 0.1% collagenase IV (Worthington, Lakewood, NJ), 0.002% DNase I (Worthington), and 0.01% hyaluronidase (Worthington) at 37 °C for 45 min. The resulting cell suspensions were filtered through a mesh filter (BD Falcon, Franklin Lakes, NJ, USA).

### Flow cytometry analysis and cell sorting

Fluorescence-activated cell sorting analysis was performed using a BD LSRFortessa™ (BD Biosciences, San Jose, CA). The following antibodies were used to classify cells: anti-CD19-fluorescein isothiocyanate (FITC) (Miltenyi Biotec, Friedrich Gladbach, Germany), anti-CD3-allophycocyanin (APC), anti-CD4-APC, anti-CD4-phycoerythrin (PE), anti-CD24-peridinin chlorophyll protein complex-Cy 5.5 (PerCP Cy5.5), anti-CD25-APC, anti-CD27-PE, anti-CD39- brilliant violet 421 (BV421), anti-CD45-V500, anti-CD73-APC, anti-IFN-γ-BV421, and anti-IL-10-BV421 (all from BD PharMingen, Franklin Lakes, NJ), anti-CD80-APC, anti-CD86-APC monoclonal antibody (mAb) (Biolegend, San Diego, CA), Annexin V- FITC (BD PharMingen), and DAPI (Cell Biolabs, San Diego, CA). The CD19^+^CD24^hi^CD27^+^ B cell subset was sorted using a Moflo XDP cell sorter (Beckman Coulter, Brea, CA) with 90% to 95% purities.

### Media

Culture medium consisting of RPMI 1640, 1% L-glutamine, 1% penicillin/streptomycin, and 10% heat-inactivated fetal calf serum (FCS) (all from Lonza) was used.

### Intracellular cytokine staining of IL-10

PBMCs were resuspended (2 × 10^6^ cells/mL) in medium and cultured in the presence of CpG (ODN M362, 10 μg/mL; Enzo Life Sciences, Farmingdale, NY) and CD40L (1 μg/mL; Miltenyi Biotec) for 24 h in 48-well flat-bottom plates. Nineteen h after stimulation, phorbol 12-myristate 13-acetate (PMA; 50 ng/mL; Wako, Osaka, Japan), ionomycin (1 μg/mL; Wako), and Golgiplug (Becton Dickinson, Franklin Lakes, NJ) were added to the culture. Twenty-four h after stimulation, cells were harvested and stained with anti-CD19-FITC, anti-CD24-PerCP Cy5.5, and anti-CD27-PE mAbs, then fixed and permeabilized with BD Cytofix/Cytoperm solution (BD Biosciences), and stained with anti-IL-10 mAb.

### Carboxyfluorescein succinimidyl ester (CFSE) labeling and proliferation assay

Cell proliferation was determined using CFSE dilution assays. CD4^+^ T cells were positively selected with anti-CD4 microbeads. For staining with CFSE (Invitrogen, Carlsbad, CA), CD4^+^ T cells at 1 × 10^6^ cells/ml in phosphate-buffered saline (PBS) were incubated with 5 μM CFSE for 10 min at 37 °C. Staining was terminated by adding RPMI 1640 containing 10% FCS at 4 °C, followed by one wash with PBS. CFSE-labeled CD4^+^ T cells were then seeded in 96-well plates (3.5 × 10^5^ cells/well; Nunc A/S, Roskilde, Denmark), and either CD19^+^CD24^hi^CD27^+^ B cells or other B cells were added at a ratio of 1:1. CpG (ODN M362, 10 μg/mL; Enzo Life Sciences), CD40L (1 μg/mL; Miltenyi Biotec), phorbol 12-myristate 13-acetate (50 ng/mL; Wako), and ionomycin (1 μg/mL; Wako) were also added to each plate. CD4^+^ T cells were profiled by CFSE labeling after 5 days of incubation. Unstained cells were included in all experiments and were used to set the compensations on the flow cytometer.

### Measurement of interferon (IFN)-γ

CD4^+^ T cells were seeded in 96-well plates (3.5 × 10^5^ cells/well; Nunc A/S). Either CD19^+^CD24^hi^CD27^+^ B cells or other B cells were added at a ratio of 1:1, and CpG and CD40Lwere added to each plate to stimulate B cells. The supernatant and cells of each well were used for enzyme-linked immunosorbent assay (ELISA) and intracellular cytokine staining of IFN-γ, respectively. For ELISA, PMA and ionomycin were added to the culture 19 h after the stimulation of CpG and CD40L. The supernatant was collected 24 h after stimulation, and IFN-γ concentrations for duplicate samples were quantified using IFN-γ Quantikine ELISA kits (R&D systems, Minneapolis, MN) following the manufacturer’s protocols. For intracellular cytokine staining of IFN-γ, PMA, ionomycin, and Golgiplug were added to the culture 19 h after the stimulation of CpG and CD40L. Twenty-four h after stimulation, cells were harvested and stained with anti-CD4-APC mAbs, then fixed and permeabilized with BD Cytofix/Cytoperm solution, and stained with anti-IFN-γ mAb.

### Apoptosis assay

CD4^+^ T cells and either CD19^+^CD24^hi^CD27^+^ B cells or other B cells were cultured in the same conditions as mentioned above. Then, the cells were collected, stained with anti-CD3-APC and anti-CD4-PE mAbs, Annexin V-FITC, and DAPI, and analyzed by flow cytometry.

### Immunohistochemical analysis

Tissue samples were fixed in formalin and embedded in paraffin. Serial sections were cut at 4 µm, dewaxed, deparaffinized in xylene, and rehydrated through a graded alcohol series. For retrieval of CD19 and IL-10, the sections were boiled for 20 min in a microwave oven in 10 mM Tris-1 ethylenediaminetetraacetic acid buffer (pH 9.0). The samples were incubated in 3% hydrogen peroxidase for 30 min to block endogenous peroxidases and in Block Ace (DS Pharma Biomedical, Osaka, Japan) for 30 min to prevent nonspecific antigen binding. The slides were subsequently incubated with primary antibodies (mouse anti-CD19 [Clone LE-CD19; DAKO, Santa Clara, CA; 1:200 dilution] and rabbit anti-IL-10 [Clone D13A11; Cell Signaling Technology, Danvers, MA; 1:50 dilution]) overnight at 4 °C. They were then incubated with alkaline phosphatase (AP)-conjugated goat anti-mouse polymer and horseradish peroxidase (HRP)-conjugated anti-rabbit polymer secondary antibodies (MACH 2 double stain®; Biocare Medical, Pacheco, CA, USA) for 30 min. Staining was visualized with HRP substrate (ImmPACT DAB®; Vector Laboratories, Burlingame, CA), which was visible as a brown color, and AP substrate (ImmPACT Vector Red®; Vector Laboratories), which was visible as a red color, and counterstained with Mayer’s hematoxylin. The presence of cells positive for CD19 and IL-10 on each slide was determined in a blinded manner. Five high-power fields were randomly selected, and the number of cells positive for both CD19 and IL-10 and those positive for CD19 in these fields was counted.

### Statistical analysis

Differences in clinicopathologic characteristics and frequency of Bregs between groups were evaluated using either paired t-tests or the Mann–Whitney *U* test. The Youden index was calculated using receiver operating characteristic (ROC) analysis to determine optimal cutoffs for Breg in the survival analysis. Overall survival (OS) was calculated according to the Kaplan–Meier method and compared using the log-rank test. Cox proportional hazards models were used for both univariate and multivariate analyses, and multivariate analysis used the factors with *P* < 0.1 in the univariate analysis. A *P* value < 0.05 was considered to indicate statistical significance. GraphPad Prism (GraphPad Software, Inc., La Jolla, CA) and Stat View (Abacus Concepts, Inc., Berkeley, CA) software were used for statistical analyses.

## Supplementary information

Suplementary Figures
