# Supplementary material for: Increased regulatory B cells are involved in immune evasion in patients with gastric cancer
Source: Sci Rep. 2019 Sep 11;9:13083. doi: 10.1038/s41598-019-49581-4 (PMC6739478; doi:10.1038/s41598-019-49581-4)
Supplement: Supplementary file 1 — Suplementary Figures [file 41598_2019_49581_MOESM1_ESM.pdf]

Increased regulatory B cells are involved in immune evasion in patients with gastric cancer

Yuki Murakami[1], Hiroaki Saito[1,*], Shota Shimizu[1], Yusuke Kono[1], Yuji Shishido[1], Kozo Miyatani[1], Tomoyuki Matsunaga[1], Yoji Fukumoto[1], Keigo Ashida[1], Tomohiko Sakabe[2], Yuji Nakayama[3] & Yoshiyuki Fujiwara[1]

[1]Division of Surgical Oncology, Department of Surgery, School of Medicine, Tottori University Faculty of Medicine, 36-1 Nishi-cho, Yonago 683-8504, Japan
[2]Division of Organ Pathology, Department of Pathology, Faculty of Medicine, Tottori University, 86 Nishi-cho, Yonago 683-8503, Japan
[3]Division of Radioisotope Science, Research Initiative Center, Organization for Research Initiative and promotion, Tottori University, 86 Nishi-cho, Yonago 683-8503, Japan

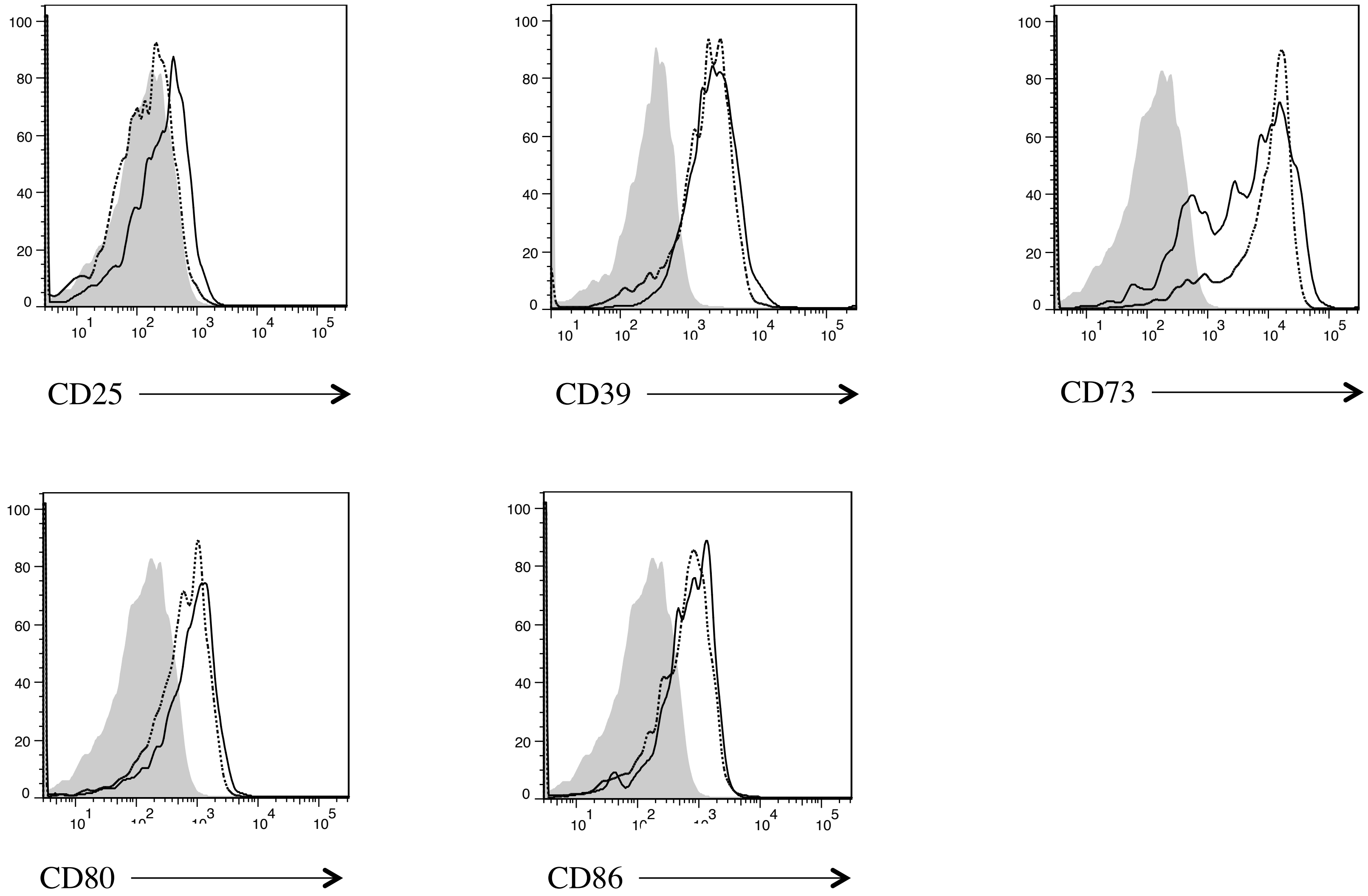

Supplementary Figure 1. A comparison of surface markers, including CD25, CD39, CD73, CD80, and CD86, between IL-10-producing B cells and non-IL-10-producing B cells. No significant difference was observed in surface marker expression between the two cell types.

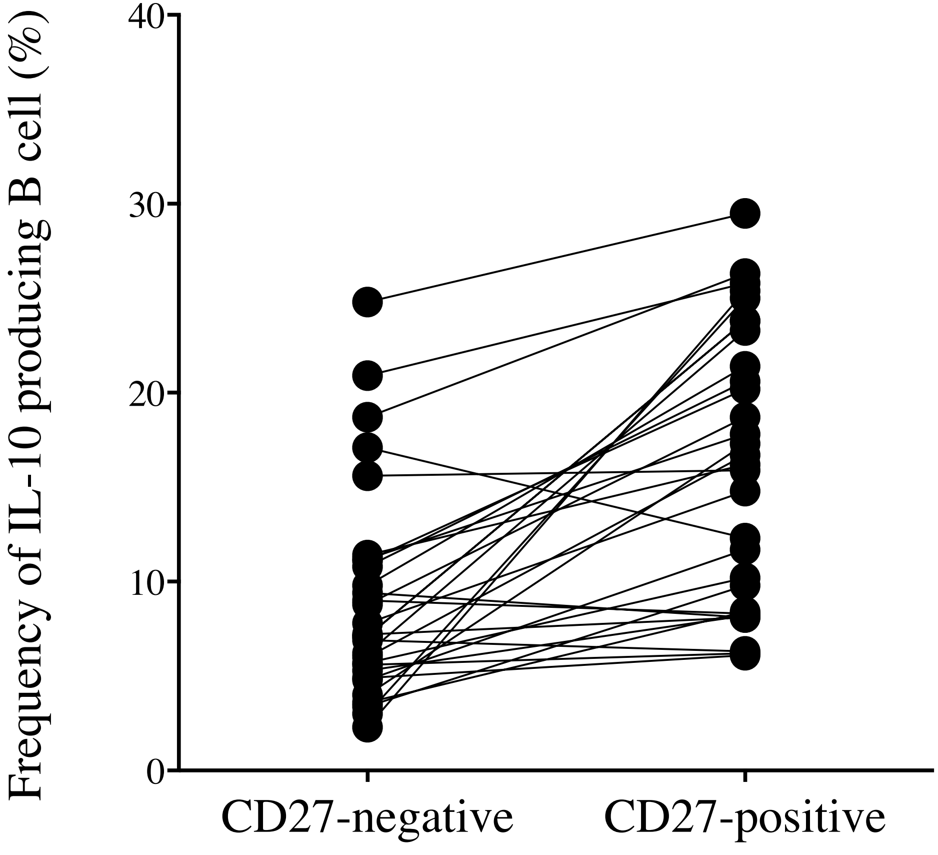

Supplementary Figure 2. IL-10-producing B cells were significantly higher in the $CD19^+CD24^+CD27^+$ B cell subset than in the $CD19^+CD24^+CD27^-$ B cell subset.

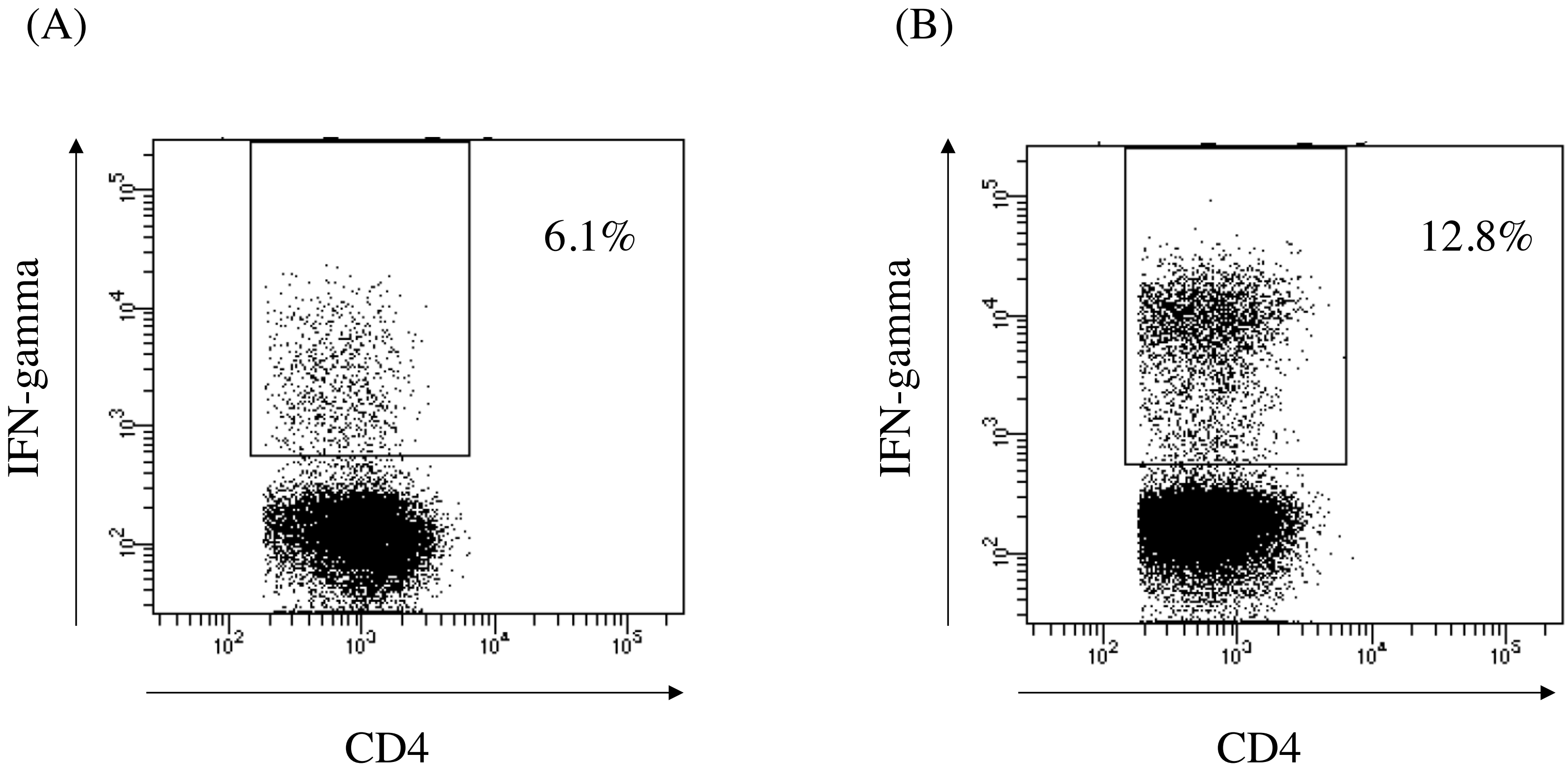

Supplementary Figure 3. Flow cytometry analysis showing that $CD19^{+}CD24^{hi}CD27^{+}$ B cells inhibited IFN-gamma production by autologous CD4+ T cells. (A) $CD4^{+}$ T cells cultured with $CD19^{+}CD24^{hi}CD27^{+}$ B cells. (B) $CD4^{+}$ T cells cultured with other B cells.

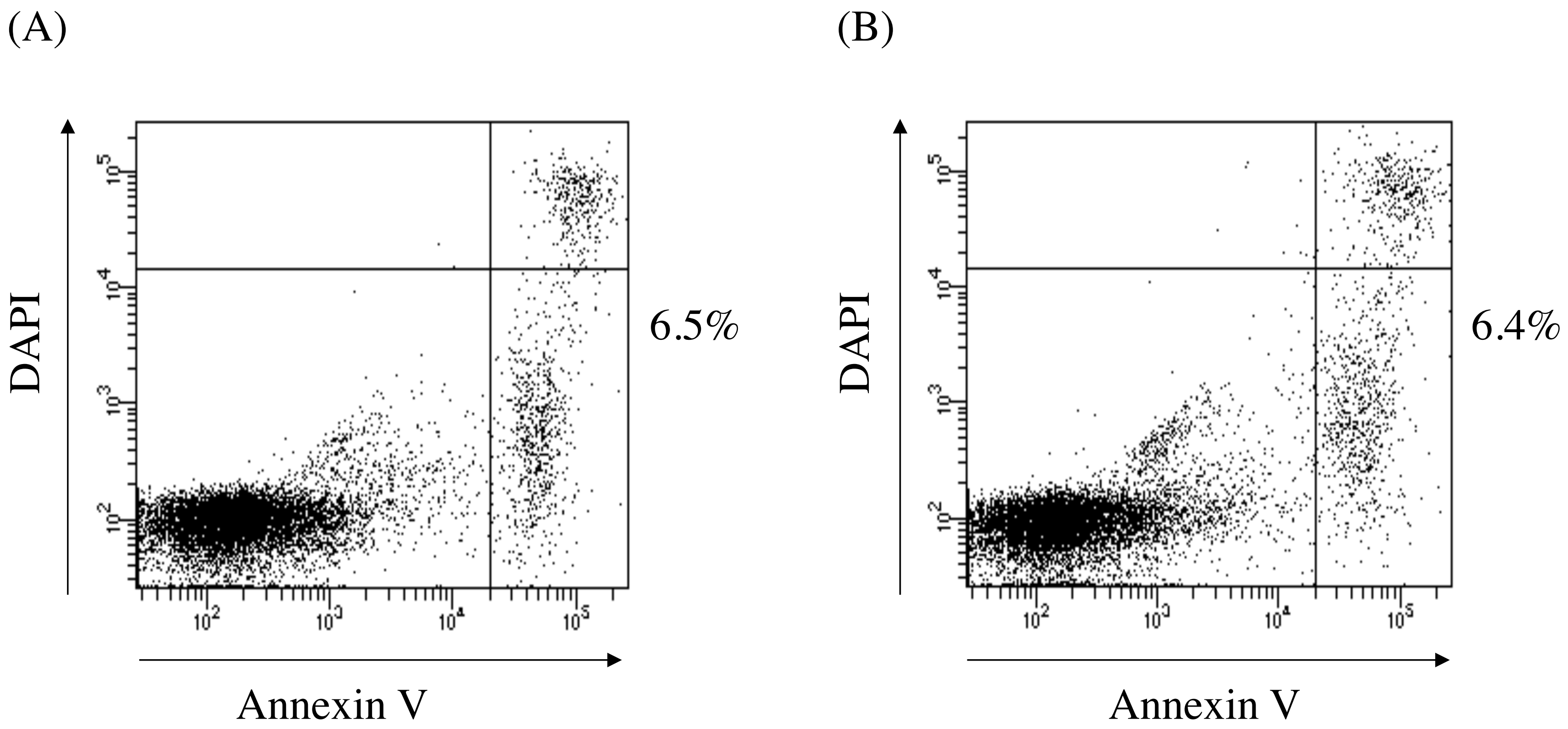

Supplementary Figure 4. Apoptosis assay showing the ratio of apoptosis of $CD4^+$ T cells. (A) $CD4^+$ T cells cultured with $CD19^+CD24^{hi}CD27^+$ B cells. (B) $CD4^+$ T cells cultured with other B cells. Percent shows the frequency of apoptosis of $CD4^+$ T cells. Cells are gated on $CD3^+CD4^+$ cells.
